# The Value of Infrared Thermography to Assess Foot and Limb Perfusion in Relation to Medical, Surgical, Exercise or Pharmacological Interventions in Peripheral Artery Disease: A Systematic Review

**DOI:** 10.3390/diagnostics12123007

**Published:** 2022-12-01

**Authors:** Giovanni Piva, Anna Crepaldi, Gladiol Zenunaj, Lorenzo Caruso, Natascia Rinaldo, Vincenzo Gasbarro, Nicola Lamberti, Pablo Jesùs Lòpez-Soto, Fabio Manfredini

**Affiliations:** 1 PhD Program in Environmental Sustainability and Wellbeing, Department of Humanities, University of Ferrara, 44121 Ferrara, Italy; 2 Unit of Nephrology, University Hospital of Ferrara, 44124 Ferrara, Italy; 3 Department of Nursing, Instituto Maimónides de Investigación Biomédica de Córdoba (IMIBIC), 14004 Córdoba, Spain; 4 Unit of Vascular and Endovascular Surgery, University Hospital of Ferrara, 44124 Ferrara, Italy; 5 Department of Environment and Prevention Sciences, University of Ferrara, 44121 Ferrara, Italy; 6 Department of Neuroscience and Rehabilitation, University of Ferrara, 44124 Ferrara, Italy; 7 Department of Nursing, Hospital Universitario Reina Sofia de Córdoba, 14004 Córdoba, Spain; 8 Department of Nursing, Pharmacology and Physiotherapy, Universidad de Córdoba, 14004 Córdoba, Spain; 9 Unit of Rehabilitation Medicine, University Hospital of Ferrara, 44124 Ferrara, Italy

**Keywords:** peripheral artery disease, infrared thermography, noninvasive, exercise, revascularization, therapy, systematic review

## Abstract

Infrared thermography (IRT) is a promising imaging method in patients with peripheral artery disease (PAD). This systematic review aims to provide an up-to-date overview of the employment of IRT as both a diagnostic method and an outcome measure in PAD patients in relation to any kind of intervention. On September 2022, MEDLINE, EMBASE, CENTRAL, Google Scholar, Web of Science, and gray literature were screened. Eligible articles employing IRT in PAD were screened for possible inclusion. The RoB 2.0 tool was used to assess the risk of bias. Twenty-one eligible articles were finally included, recruiting a total of 1078 patients. The IRT was used for PAD diagnosis/monitoring in 11 studies or to assess the effect of interventions (revascularization, pharmacological therapy, or exercise rehabilitation) in 10 studies. The analysis of the included papers raised high concerns about the overall quality of the studies. In conclusion, IRT as a noninvasive technique showed promising results in detecting foot perfusion in PAD patients. However, limits related to devices, points of reference, and measurement conditions need to be overcome by properly designed trials before recommending its implementation in current vascular practice.

## 1. Introduction

Peripheral arterial disease (PAD) is a highly prevalent progressive disease that affects millions of people worldwide [1]. The symptoms of PAD are caused by insufficient arterial blood flow to the lower extremities due to artery damage by aging, obesity, smoking, heart conditions, diabetes, family history, hypertension, or hyperlipidemia that occurs throughout the entire body [2]. In the lower limbs, reduced oxygen delivery results in ischemia-induced, debilitating leg discomfort associated with walking in the intermediate stages or with critical rest pain or foot ulcers in the advanced stages of the disease [3].

The diagnosis of PAD is composed of physical examination and diagnostic testing. The diagnostic procedures included measurements of the ankle-brachial index, segmental lower extremity blood pressures, duplex ultrasound, computed tomography angiography, magnetic resonance angiography, and invasive angiography [1].

In addition, other methods are employed to determine foot perfusion, including the toe-brachial index, transcutaneous oxygen pressure or skin perfusion pressure, capillaroscopy, and near-infrared spectroscopy (NIRS)-assisted tests [1,4,5]. All these techniques have strengths and weaknesses. First, the toe-brachial index is used to establish a diagnosis of PAD or to assess perfusion in patients with critical perfusion when ABI is not reliable, but it has limitations in terms of specificity and diagnostic criteria [6,7]. Additionally, transcutaneous oxygen pressure is a valid and available tool capable of detecting skin rather than tissue perfusion pressure, with a long collection and implementation time [8]. Finally, NIRS has also been employed in studies on PAD patients in static and dynamic conditions for noninvasive assessment of muscle metabolism. This technique monitors the local balance between oxygen delivery and consumption and provides information on tissue microvascular hemodynamics [4,9,10,11,12]. Being easily usable also for bedside measurements, the technique was tested in PAD for diagnostic purposes as well for foot perfusion assessment under dynamic conditions or in response or during therapeutic treatments [5,13,14,15,16,17,18,19].

Recently, infrared thermography (IRT) has emerged as a promising imaging method since it can detect the infrared light emitted by the body and visualize body temperature changes related to blood flow [20]. Infrared thermography is an instrumental tool that collects infrared radiation radiated by the human body through an infrared thermal imaging camera lens and converts it into temperature heat by photoelectric conversion.

Infrared radiation is a component of electromagnetic radiation, which is emitted by every object at temperatures above zero degrees. This radiation pertains in equal proportion to the intrinsic temperature of the item. The beams of the radiation emitted by the object are focused on a detector element through the optics in the thermographer. These electrical signals are said to be proportional to the radiation. These signals are then analyzed and blown out to generate output signals that depict the temperature directly. The data received can surface on a computer screen or be sent to a control system via a connecting interface. The measurement of infrared temperature is based on the law of Planck’s radiation. It is directly correlated to the spectral radiation of a black body into space depending on its temperature and wavelength.

This technique is noncontact, noninvasive, low cost, and allows a quick examination of the energy radiated by the user’s body. It is safe for both patients and health professionals and is reliable for the measurement of skin temperature [21,22,23].

Several systematic reviews have been conducted aiming at assessing the use of IRT in inflammatory diseases [24], tendinopathies [25], burns [26], temporomandibular disorders [27], and in the oncology field [28].

To the best of our knowledge, just one systematic review has investigated tissue perfusion techniques and their clinical values in patients with PAD [20], but the authors included only one trial assessing plantar thermography, and a narrative review published 10 years ago investigated the use of IRT in the medical fields [29].

Therefore, this systematic review aims to provide a comprehensive overview of the different instrumentations and methods of employing IRT and to investigate its effectiveness as a diagnostic tool for the detection and assessment of any interventional procedure in patients affected by PAD at any severity.

## 2. Materials and Methods

This systematic review was conducted in accordance with the Preferred Reporting Items for Systematic reviews and Meta-Analysis (PRISMA) guidelines [30]. Eligible articles were included if they described the use of IRT to determine foot temperature and/or tissue perfusion in adult patients with PAD at any stage and in relation to medical, surgical, exercise, or pharmacological interventions. The included articles were full-text articles published between 1 January 1990 and 30 September 2022, without any language restriction.

### 2.1. Literature Search

The following electronic bibliographic databases were searched for eligible articles: MEDLINE, EMBASE, The Cochrane Library (Cochrane Database of Systematic Reviews, Cochrane Central Register of Controlled Trials (CENTRAL), Cochrane Methodology Register), Google Scholar and Web of Science (science and social science citation index). The gray literature has been screened through research from institutional repositories or online platforms.

The search strategy included medical subject headings (MeSH) terms for “peripheral arterial disease”, “peripheral vascular diseases” and words for “temperature”, “thermograph*”, and “perfusion”. The complete search strategy is available in Supplementary Material. The search terms have been adapted for use with other bibliographic databases.

The titles and abstracts of the studies were independently screened by two authors (G.P. and A.C.) who were blinded to the study authors and journal titles. Articles considered for inclusion were independently reviewed by the same two authors. Disagreements were resolved by discussion or by consensus after consulting a third reviewer (N.L.).

### 2.2. Data Collection

Two reviewers (G.P. and A.C.) independently extracted the data on a properly developed electronic spreadsheet. The data collected included publication details (first author, year, country), study design, participants’ characteristics (age, sex, sample size, PAD severity), inclusion and exclusion criteria, sampling process, reference values, and all measures related to precision, outcomes and time of measurements, interventions, control conditions, indicators of acceptability to users and possible outcomes (temperature variations after any type of intervention).

The Risk of Bias tool 2.0 was used by two independent reviewers (N.L. and F.M.) to assess the risk of bias and applicability of the studies [31]. This tool was used to assess the risk of bias in patient selection, blindness, and randomization, and a three-way classification (Low, Some Concerns, High risk for 5 domains) was provided for each included study.

## 3. Results

**Figure 1:**
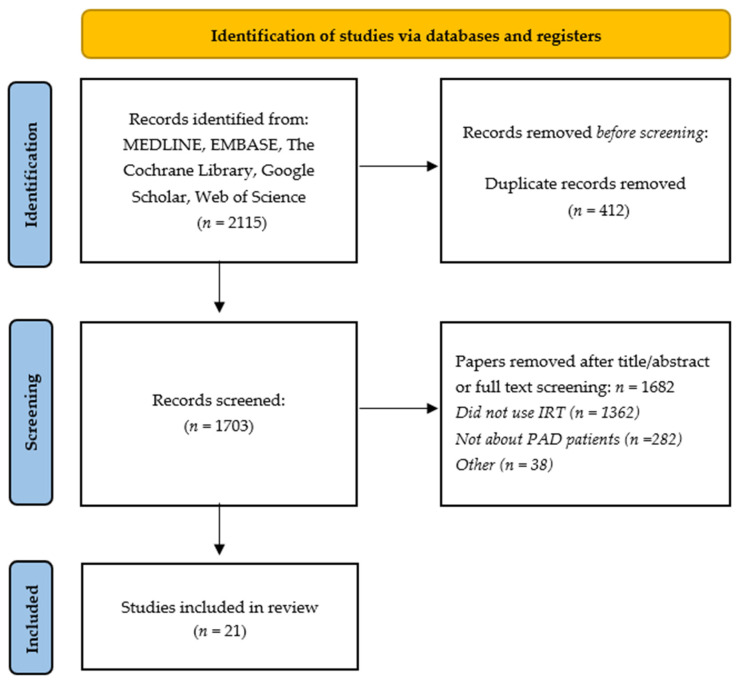
Study flow diagram (PRISMA 2009).

The database searches resulted in 2115 identified records, of which 412 were duplicates. After title and abstract screening, 1630 articles were excluded according to the inclusion criteria. The full texts of the remaining 73 papers were screened, leading to the exclusion of 52 articles. Finally, 21 articles were found to be eligible for inclusion. The study flow diagram is shown in Figure 1.

The included studies mainly employed IRT devices manufactured in the United States (*n* = 16), while three trials used Japanese devices, and the remaining two studies used thermal cameras made in Taiwan. The characteristics of the thermal cameras are reported in Table 1.

### 3.1. Included Studies

The characteristics of the included studies are reported in Table 2. Some differences are present in relation to the number of subjects recruited (*n* = from 2 to 257) and the age of the participants included (28 to 72 years). (Table 2).

The risk of bias was high overall, since only one trial was randomized, and possible bias may have arisen from the inclusion of the subjects. Otherwise, in relation to the quality of the measurements and the missing outcome, the risk of bias was found to be low for the majority of the studies included. The analysis of the risk of bias is reported in Supplementary Material Figure S1.

### 3.2. Measurement Locations

**Figure 2:**
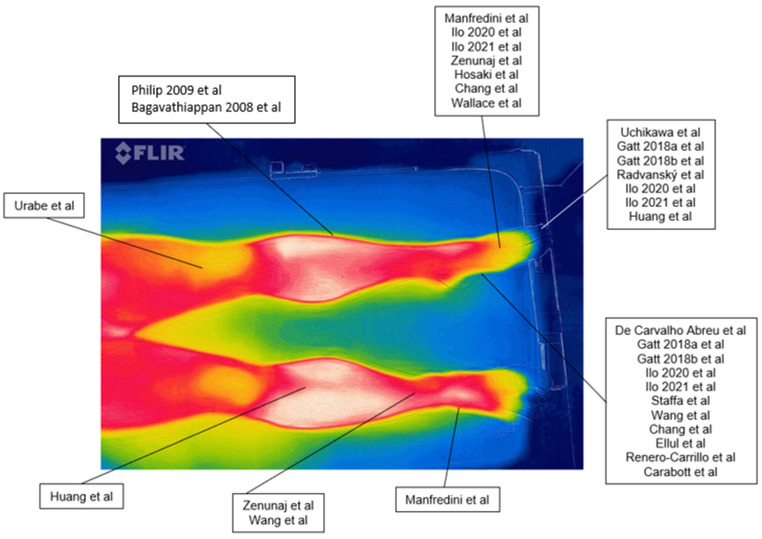
Example of a thermographic image with the site of the collection according to each study. References: [2,21,22,32,33,34,35,36,37,38,39,40,41,42,43,44,45,46,47,48,49].

The included studies reported different measurement sites, focusing in particular on the feet. One study did not precisely describe the measurement location [45], 2 articles studied the ankle and the anterior tibial face [39,44], six the toes [2,32,33,41,42,43,48], seven the dorsum of the foot [31,32,37,38,39,46,49] and eleven authors identified the plantar side of the foot, making it the most studied area [22,32,33,34,35,36,40,42,44,50]. A summary of the measurement locations is reported in Figure 2.

### 3.3. Diagnosis of PAD and Assessment of Peripheral Perfusion

Five studies aimed to use IRT as a diagnostic tool for the detection of PAD [2,21,32,47,48]. Radvanský et al. proposed utilizing IRT as a low-cost device to identify the occurrence of PAD in toes [2]. Bagavathiappan et al. [47] and Philip et al. [21] published two articles with this aim, exploiting the noninvasiveness of the IRT technique and observing a temperature difference of approximately 0.7–1 °C between the affected and nonaffected limbs. The study of Ilo et al. [32] compared IRT with the measurement of the ankle-brachial index (ABI); the authors concluded that IRT alone cannot be recommended for evaluating PAD because normal skin surface temperature varies among individuals; however, it does have the potential to provide additional information about circulation, infections, and the severity of the disease. Finally, Huang et al. [48] used IRT in the evaluation of a cohort of healthy and PAD patients by measuring the temperature before and after a walking test and detecting a different temperature change in the feet between PAD and non-PAD patients (−1.25 °C vs. −0.15 °C).

Four studies considered IRT as an effective tool for the evaluation of peripheral perfusion. Wang et al. [44] used IRT to study blood circulation by comparing the temperature of the hands, legs, and feet of PAD patients and observing large temperature differences (>10 °C) at the foot, especially the toes. The authors concluded that IRT could potentially be a very valuable tool for vascular diseases, especially thanks to the information obtained from the temperature distribution maps. Hosaki et al. [46] focused their study on patients with diabetes mellitus (DM) and PAD at early stages, finding that IRT could be useful as a noninvasive method to timely identify patients with poor peripheral circulation and for the evaluation of further treatment. Gatt et al. [41] used IRT to investigate where heat emitted from the foot of the patients differed in patients affected by DM, with or without PAD. The authors observed a significant difference in temperature at all the toes, with patients affected by concomitant DM and PAD showing a higher temperature than patients with DM only.

Gatt et al. [42] evaluated the potential of IRT for the detection of foot complications in patients with DM. The participants in the study were divided into five groups: healthy adults, DM with no complications, DM with peripheral neuropathy, DM with neuroischemia, and DM with PAD. The authors reported that the temperature of the toes of patients with complications was higher than that of the groups of healthy adults and patients with DM without complications, concluding that the higher the temperature was, the higher the probability that a patient could be affected by PAD, neuropathy or neuroischemia.

The study of de Carvalho Abreu et al. [22] correlated IRT findings and ABI in patients with PAD and observed a strong direct correlation between IRT and ABI in patients with noncalcified arteries. Moreover, the study of Wallace et al. [37] found a positive correlation, concluding that IRT is a valid method in addition to ABI to assess PAD.

### 3.4. Assessment after Different Interventions

Five studies have monitored the effect of revascularization procedures using IRT [33,34,35,39,49]. Zenunaj et al. [39] evaluated the effectiveness of an intervention by measuring the temperature in both feet before and 24 h after the surgery, observing an improvement of +2.1 °C and +0.5 °C in the limb that underwent the revascularization. Similarly, the article of Staffa et al. [34] evaluated IRT as a supplementary method in assessing the treatment effect of percutaneous transluminal angioplasty by obtaining thermal images before and after the surgery. The median temperature change in the treated leg was +0.4 °C, and for the nontreated leg, it was −0.5 °C.

Additionally, Ilo et al. [33] compared skin temperature changes in feet before and after different methods of vascular surgeries, showing a median change of +0.5 °C on the revascularized side, and −0.3 °C on the nontreated side. Renero-Carrillo et al. [35] reported a case report comparing the IRT images of a woman before and after revascularization surgery with those of a woman with no signs or symptoms of PAD. While measurements on the healthy subject remained constant, the temperature in the treated foot increased by 6 °C and decreased in the nontreated foot by −0.7 °C.

Finally, the study of Chang et al. [49] explored the feasibility of IRT to assess reperfusion of the foot after endovascular therapy and to predict amputation in patients with critical limb ischemia by using IRT before and after the procedure. Both wound healing and freedom from major amputation during the 6-month follow-up period were recorded. The authors revealed that the mean pre- and postintervention temperature of the foot was significantly higher in the group that had healed ulcers at 6 months than in nonhealing patients (30.7 °C vs. 29.4 °C, and 32.3 °C vs. 30.9 °C), while the variations in temperature after revascularization were similar (+1.5 °C). The authors concluded that IRT was associated with wound healing and helped to predict freedom from major amputation.

Two studies have used IRT to assess variations in temperature after a pharmacological treatment [43,45]. Urabe et al. [45] administered nafamostat, a serine inhibitor, to determine if it could improve local hemodynamics under ischemic conditions caused by exercise in patients with PAD. The authors reported that IRT detected an improvement of 0.2 °C in the skin temperature of the affected legs after the treatment. The study of Uchikawa et al. [43] investigated the effect of cilostazol, a selective inhibitor of platelet cAMP-phosphodiesterase, on PAD in patients with DM. The authors analyzed the temperature of the toes before and after drug administration and detected an average increase of 3.3 °C in the two legs and of 4.4 °C in the lower foot.

The study of Manfredini et al. [38] will compare the effects of a low-intensity, pain-free structured home-based exercise program to a control group that will be advised to walk, according to guidelines, on foot temperature by assessing four temperature points at the ankle and the foot dorsum. Since the authors published the study protocol, no results are yet available.

The aim of the study of Ellul et al. [36] was to explore the effect of calf muscle electrostimulation on peripheral perfusion, measured with IRT, in patients with DM and PAD. At the end of the 12-week program, the authors detected a significant increase in the resting temperature of all the regions of interest (mean increase of +2.4 °C).

The study of Carabott et al. [40] aimed to compare temperature changes following a challenge of limb elevation using IRT. The participants were categorized into a no PAD, mild PAD, or severe PAD group. All patients underwent thermal imaging, and successive thermal images were taken at one-minute intervals after the lower limbs were elevated for five minutes. Thereafter, the lower limbs were lowered to the original position and imaged after one minute. The authors found that the mean resting temperatures of all angiosomes of the PAD group were higher than those with no PAD.

## 4. Discussion

The systematic review included studies that employed IRT in patients with PAD as both diagnostic methods, or as an outcome for measuring the success of an interventional procedure. At first, IRT demonstrated its versatility, being employed in studies enrolling PAD patients at any disease stage and being exposed to different procedures, including revascularization [33,34,35,39,49], pharmacological [43,45] or physical [36,38].

In relation to the twenty-one included studies, the majority of them (*n* = 13) had a relatively small sample size (less than 50 patients), while the remaining eight recruited a significant number of participants (from 51 to 217). In addition, the studies enrolled patients with a wide heterogeneity of age, from relatively young age patients (mean age < 40 years old [47] to older patients (mean age > 75 years [39]), making it difficult to summarize the data collected.

Despite the heterogeneity of the population enrolled and of the data collected, all authors agreed on the utility of IRT in the monitoring of PAD patients, and particularly Carvalho Abreu et al. [22] suggest preferring IRT to ABI in patients who did not appreciate the latter technique, with these two measurements strictly correlated. Although the use of IRT as a diagnostic technique is still debated, more evidence is provided when employing IRT as an outcome measure. Several manuscripts used IRT to detect the effects of different revascularization procedures, pharmacological therapies, or exercise interventions concerning variations in temperature and heat distribution across the foot. To this end, incongruence was found between the majority of the included studies and two manuscripts from Gatt et al. [42,43]. In particular, all authors reported that the lower the foot temperature was, the more severe PAD was, in contrast to the finding of Gatt et al., who observed that patients with PAD had a higher foot temperature than patients without PAD [42,43].

Generally, considering the results of the 21 eligible studies, which were mostly affected by some or high concerns, reflecting poor quality according to the RoB-2 tool, these are not sufficient to fully demonstrate IRT implementation in clinical practice, even if De Deus Passos and Ferreira Da Rocha recently published promising results in terms of the sensitivity and specificity of IRT when compared to color Doppler ultrasonography [51]. Non-randomized or properly designed diagnostic trials are presented in this review, and several biases and limitations need to be addressed.

At first, many different IRT devices are available on the marketplace, each with personal characteristics and different resolutions, spectral ranges, standard temperature ranges, prices, and accuracies. Particularly, the majority of the devices report a significant margin for error, considering that ±2 °C is a significant percentage of the skin or body temperature and that the improvement after revascularization was reported between 1–3 °C. Moreover, many different points of reference where the measurement was taken are reported, from the shin to the ankle or covering the foot dorsum, the toes, or the foot plant. Another issue to be addressed is the different measurement conditions, in particular, the room temperature and the time of exposure barefoot to the room temperature. Indeed, it is easy to imagine that the temperature of an operating room is lower than that of an outpatient clinic and that a measurement carried out after 1 or 5 min of bed rest could lead to different results. Finally, we must bear in mind that because normal skin surface temperature varies among individuals, IRT alone cannot be recommended for diagnosing PAD because it is impossible to determine a validated cutoff value [32].

On the other hand, IRT has significant advantages that make it very interesting for its employment in PAD patients, considering its low cost compared to other diagnostic techniques, its ease-of-use, and fast-collecting procedure, and its reliability and repeatability of the measurements taken. All these promising features need to be exploited by involving producers, researchers, and clinical professionals in reducing the margin of error of the devices, designing proper trials investigating their diagnostic accuracy, collecting outcomes in a standardized way, and defining reference points of collection, time, and measurement conditions.

## 5. Conclusions

In conclusion, this is the first systematic review exclusively investigating the use of IRT in PAD patients. This noninvasive technique showed promising results in the screening of PAD and the detection of different areas of foot perfusion, and it provided satisfactory results in measuring the effects of any kind of intervention. Otherwise, despite favorable opinions and considering the importance of the investigation of tissue perfusion, the evidence remains low regarding diagnostic accuracy or IRT, and it is too early to recommend it as the only decision tool in the assessment of patients with PAD. Properly designed, prospective observational and interventional studies with a certain length of follow-up are necessary to recommend the implementation of IRT in current vascular practice.

## Figures and Tables

**Table 1:** The different models of thermography devices employed in the included studies.

Model	Resolution	Spectral Range	Standard Temperature Range	Accuracy	Reference
FLIR A325sc	320 × 240	7.5–13 μm	−20 °C to 120 °C; 0 °C to 350 °C	±2 °C or ±2% of reading	[32,33]
FLIR B200	1280 × 1024	7.5–13 μm	−20 °C to 120 °C; up to 1200 °C	±2 °C or ±2% of reading	[34]
FLIR E6	320 × 240	7.5–13 μm	−20 °C to 250 °C	±2 °C or ±2% of reading	[35]
FLIR I3	60 × 60	7.5–13 μm	−20 °C to 250 °C	±2 °C or ±2% of reading	[36]
FLIR Lepton 3.5	160 h × 120 v	8–14 μm	−10 °C to 140 °C; −10 °C to 400 °C; −10 °C to 450 °C	±5 °C or 5%; ±10 °C or 10%	[2]
FLIR ONE-Pro	1440 × 1080	8–14 µm	−20 °C to 400 °C	±3 °C o ±5%	[37,38,39]
FLIR SC630	NA	NA	NA	NA	[40]
FLIR SC7200	320 × 256	1.5–5.1 µm	−20 °C to 300 °C; 5°C to 1500 °C; up to 2500 °C	±1 °C or ±1% of reading	[41,42]
FLIR T430SC	320 × 240	7.5–13 μm	−20 °C to 120 °C; 0 °C to 650 °C	±2 °C or ±2% of reading	[22]
Infra-eye-150	NA	NA	NA	NA	[43]
Raytheon Radiance HS	256 × 256	3–5 µm	NA	NA	[44]
Thermo Tracer 6T66	NA	NA	NA	NA	[45,46]
Thermovision 550	NA	3.6–5 µm	−20 °C to 250 °C, up to 1500 °C	±2 °C or ±2% of reading	[21,47]
Spectrum 9000-MB Series	320 × 240	NA	NA	NA	[48,49]

Abbreviations: NA, not available.

**Table 2:** The different models of thermography devices employed in the included studies.

Study (Year)	Study Design	Scope of the Study	PAD Stage	Sample Size	Age	Measurement Location	Temperature Variations	Other Outcomes	Reference
Bagavathiappan et al. (2008)	Case report	Detection of PAD	NA	3	35.7 (28–48)	Shins, Feet	NA	NA	[47]
Philip et al. (2009)	Technical note	Detection of PAD	NA	4	36.7 (28–40)	Shins, Feet	NA	NA	[21]
Carabott et al. (2021)	Observational study	Assessment after limb elevation challenge	I–III	27	62.7 ± 6 non-PAD; 71.1 ± 7 mild PAD; 71 ± 5 severe PAD	Feet	−0.03 °C (non-PAD) −0.18 °C (mild PAD) −0.27 °C (severe PAD)	NA	[40]
Chang et al. (2020)	Observational study	Assessment after revascularization	IV	124	73 (63–80)	Feet	+1.3 °C (healing) +1.3 °C (nonhealing)	NA	[49]
de Carvalho Abreu et al. (2022)	Nonrandomized	Correlation IRT-ABI	I–IV	53	59.9 ± 16.3	Feet	NA	ABI	[22]
Ellul et al. (2017)	Randomized	Assessment after EMS	II	40	70.8 ± 7	Feet	−0.5 °C (baseline) −0.3 °C (follow-up)	ABI, ACD	[36]
Gatt et al. (2018)	Nonrandomized	Assessment of peripheral perfusion	III	53	64.5 ± 5 females DM + PAD; 72.2 ± 8 males DM + PAD; 59.8 ± 11 females DM; 65.8 ± 7 males DM	Feet	NA	ABI, SD	[41]
Gatt et al. (2018)	Nonrandomized	Detection of foot complications	I–II	182	NA	Feet	NA	ABI, SD, 10 g monofilament	[42]
Hosaki et al. (2002)	Case report	Assessment of peripheral perfusion	NA	27	67.4 (51–82)	Feet	NA	LDBF	[46]
Huang et al. (2011)	Observational study	Detection of PAD	NA	51	72.9 ± 10 PAD; 68.2 ± 8 non-PAD	Shins, Feet	−1.25 °C (PAD) −0.15 (non-PAD)	6MWD, ABI, SBP	[48]
Ilo et al. (2020)	Observational study	Detection of PAD	I–II	257	73 ± 10 PAD; 61 ± 18 non-PAD	Feet	NA	ABI, TP	[32]
Ilo et al. (2021)	Nonrandomized	Assessment after revascularization	I–III	54	72 ± 10 treated;75 ± 8 control	Feet	+0.5 °C (treated) −0.3 °C (nontreated) −0.2 °C (control)	ABI, TP	[33]
Manfredini et al. (2021)	Study protocol	Assessment after exercise program	II	60	>60	Feet	NA	6MWD, ABI, QoL, 5TSTS	[38]
Radvanský et al. (2022)	Commentary	Detection of PAD	NA	NA	NA	Feet	NA	NA	[2]
Renero-Carrillo et al. (2021)	Nonrandomized	Assessment after revascularization	NA	2	60.5 (55–66)	Feet	+6 °C (treated) −0.7 °C (nontreated)	PPG	[35]
Staffa et al. (2016)	Nonrandomized	Assessment after revascularization	I–III	41	60.2 ± 18 PAD; 55.6 ± 18 non-PAD	Feet	+0.4 °C (treated) −0.5 °C (nontreated)	ABI	[34]
Uchikawa et al. (1992)	Observational study	Assessment after pharmacological therapy	II	10	51 (36–68)	Feet	+3.3 °C (average) +4.4 °C (lowest)	NA	[43]
Urabe et al. (1993)	Randomized	Assessment after pharmacological therapy	II	20	65.6 ± 2.3	Legs	+0.2 °C	Walking distance, Lac, PPG	[45]
Wallace et al. (2018)	Case report	Assessment of peripheral perfusion	I-IV	23	66 (30–76)	Feet	NA	ABI	[37]
Wang et al. (2004)	Observational study	Assessment of peripheral perfusion	NA	7	NA	Ankle, Feet	NA	NA	[44]
Zenunaj et al. (2021)	Observational study	Assessment after revascularization	II-IV	40	76 ± 11	Ankle, Feet	+2.1 °C (lowest) +0.5 °C (highest)	ABI	[39]

PAD: peripheral arterial disease; Lac: lactate levels; PPG: plethysmography; IRT: infrared thermography; ABI: ankle-brachial index; 6MWD: six-minute walking distance; QoL: quality of life; 5TSTS: 5-times sit-to-stand test; NA: not available; SD: spectral Doppler; TP: toe pressure; LDBF: laser Doppler blood flowmetry; SBP: segmental blood pressure; EMS: muscle electro stimulation; ACD: absolute claudication distance; DM: diabetes mellitus.

## Data Availability

Not applicable.

## Supplementary Materials

The following supporting information can be downloaded at https://www.mdpi.com/article/10.3390/diagnostics12123007/s1, Figure S1: Risk of bias of the included studies.

## Registration

CRD42022325281 (registered on 10 May 2022).
