# Supplementary material for: The Value of Infrared Thermography to Assess Foot and Limb Perfusion in Relation to Medical, Surgical, Exercise or Pharmacological Interventions in Peripheral Artery Disease: A Systematic Review
Source: Diagnostics (Basel). 2022 Dec 1;12(12):3007. doi: 10.3390/diagnostics12123007 (PMC9777328; doi:10.3390/diagnostics12123007)
Supplement: Supplementary file 1 [file diagnostics-12-03007-s001.zip › diagnostics-2012837-supplementary.pdf]

The Value of Infrared Thermography to Assess Foot and Limb Perfusion in Relation to Medical, Surgical, Exercise or Pharmacological Interventions in Peripheral Artery Disease: A Systematic Review

Giovanni Piva † , Anna Crepaldi †, Gladiol Zenunaj, Lorenzo Caruso , Natascia Rinaldo, Vincenzo Gasbarro, Nicola Lamberti, Pablo Jesùs Lòpez-Soto and Fabio Manfredini

† These authors contributed equally to this work.

Supplementary Figure S1. Risk of bias determined by RoB 2.0 Tool for all the studies included in the review.

Study ID	D1	D2	D3	D4	D5	Overall
Bagavathiappan 2008	-	!	+	+	!	-
Bagavathiappan 2009	-	!	+	+	!	-
Carabott 2021	-	!	+	+	!	-
Chang 2020	-	+	+	+	!	-
de Carvalho Abreu 2022	-	+	+	+	!	-
Ellul 2017	-	!	+	+	!	-
Gatt 2018	-	!	+	+	!	-
Gatt 2018	-	+	+	+	!	-
Hosaki 2002	-	+	+	+	!	-
Huang 2011	-	+	+	+	!	-
Ilo 2020	-	+	+	+	!	-
Ilo 2021	-	+	+	+	!	-
Manfredini 2021	+	+	+	+	!	!
Radvanský 2022	-	+	+	+	!	-
Renero-Carrillo 2021	-	+	+	+	!	-
Staffa 2017	-	!	+	+	!	-
Uchikawa 1992	-	!	+	+	!	-
Urabe 1993	-	!	+	+	!	-
Wallace 2018	-	+	+	+	!	-
Wang 2004	-	+	+	+	!	-
Zenunaj 2021	-	!	+	+	!	-

+ Low risk
! Some concerns
- High risk

D1 Randomisation process
D2 Deviations from the intended interventio
D3 Missing outcome data
D4 Measurement of the outcome
D5 Selection of the reported result
